# Contrasting allelic distribution of *CO*/*Hd1* homologues in *Miscanthus sinensis* from the East Asian mainland and the Japanese archipelago

**DOI:** 10.1093/jxb/erv292

**Published:** 2015-06-18

**Authors:** Hironori Nagano, Lindsay V. Clark, Hua Zhao, Junhua Peng, Ji Hye Yoo, Kweon Heo, Chang Yeon Yu, Kossonou Guillaume Anzoua, Tomoaki Matsuo, Erik J. Sacks, Toshihiko Yamada

**Affiliations:** ^1^Field Science Center for Northern Biosphere, Hokkaido University, Sapporo, Hokkaido 060-0810, Japan; ^2^Department of Crop Sciences, University of Illinois, Urbana-Champaign, Urbana, IL 61801, USA; ^3^College of Horticulture and Forestry Science, Huazhong Agricultural University, Wuhan, Hubei 430070, China; ^4^Science and Technology Center, China Seed Group Co. Ltd, Wuhan, Hubei 430206, China; ^5^Kangwon National University, Chuncheon, Gangwon 200-701, South Korea

**Keywords:** Bioenergy, flowering time gene, gene duplication, geographical allelic differentiation, *Miscanthus sinensis*, MITEs.

## Abstract

The genus *Miscanthus* is a perennial C_4_ grass native to eastern Asia and is a promising candidate bioenergy crop for cool temperate areas. Flowering time is a crucial factor governing regional and seasonal adaptation; in addition, it is also a key target trait for extending the vegetative phase to improve biomass potential. Homologues of *CONSTANS (CO*)/*Heading date 1(Hd1*) were cloned from *Miscanthus sinensis* and named *MsiHd1*. Sequences of *MsiHd1* homologues were compared among 24 wild *M. sinensis* accessions from Japan, 14 from China, and three from South Korea. Two to five *MsiHd1* alleles in each accession were identified, suggesting that *MsiHd1* consists of at least three loci in the *Miscanthus* genome. Verifying the open reading frame in *MsiHd1*, they were classified as putative functional alleles without mutations or non-functional alleles caused by indels. The Neighbor–Joining tree indicated that one of the multiple *MsiHd1* loci is a pseudogene locus without any functional alleles. The pseudogene locus was named *MsiHd1b*, and the other loci were considered to be part of the *MsiHd1a* multi-locus family. Interestingly, in most Japanese accessions 50% or more of the *MsiHd1a* alleles were non-functional, whereas accessions from the East Asian mainland harboured only functional alleles. Five novel miniature inverted transposable elements (MITEs) (*MsiMITE1*–*MsiMITE5*) were observed in *MsiHd1a/b*. *MsiMITE1*, detected in exon 1 of *MsiHd1a*, was only observed in Japanese accessions and its revertant alleles derived from retransposition were predominantly in Chinese accessions. These differences in *MsiHd1a* show that the dependency on functional *MsiHd1a* alleles is different between accessions from the East Asian mainland and Japan.

## Highlight

Homologues of *CONSTANS*/*Heading date 1* were cloned from *Miscanthus sinensis* and named *MsiHd1. MsiHd1a* in most Japanese accessions contained non-functional alleles, whereas Asian mainland accessions harboured only functional alleles.

## Introduction

The genus *Miscanthus* is a rhizomatous perennial C_4_ grass that grows naturally in East Asia and Oceania (Hodkinson *et al.*, 2002). *Miscanthus* spp. are now a leading choice for bioenergy crops in cool temperate regions. Taxonomically, *Miscanthus* belongs to the Andropogoneae tribe along with maize (*Zea mays* L.), sorghum (*Sorghum bicolor* (L.) Moench), and sugarcane (*Saccharum* hybrids) (Hodkinson *et al.*, 2002). Among *Miscanthus* spp., *Miscanthus sinensis* Anderss., *Miscanthus sacchariflorus* (Maxim.) Franch. and *Miscanthus*×*giganteus* Greef & Deuter ex Hodkinson & Renvoize have gained attention as bioenergy crop candidates. *M. sinensis* is naturally distributed in East and South-East Asia and it is typically diploid (Lafferty and Lelly, 1994; Sacks *et al.*, 2013). *M. sacchariflorus* is also native to East Asia, and includes diploid and tetraploid ecotypes. Most *M. sacchariflorus* in China are diploid, whereas *M. sacchariflorus* in Japan is predominantly tetraploid. To date, commercial biomass production using *Miscanthus* is limited to the cultivation of a single clone of *M.*×*giganteus*, a sterile interspecific hybrid between *M. sinensis* and *M. sacchariflorus* that was brought from Yokohama, Japan to Denmark in the 1930s by Aksel Olsen (Greef and Deuter, 1993; Linde-Laursen, 1993).

*M. sinensis* has been studied as a bioenergy crop alternative to *M.*×*giganteus* because of its greater cold tolerance and yield in cold regions, ability to be propagated by seed, the availability of genetic resources, and the possibility to develop new genotypes through conventional breeding programmes (Stewart *et al.*, 2009; Hodgson *et al.*, 2010; Anzoua *et al.*, 2015). *M. sinensis* is adapted primarily to environments that have an average annual minimum temperature of –28.9 °C or greater (USDA hardiness zone 5 or warmer) and receive ≥750mm of precipitation annually (Clifton-Brown *et al.*, 2008; Sacks *et al.*, 2013). *M. sinensis* is an early colonizer after ecological disturbance in environments that would otherwise support forest (Numata and Mitsudera, 1969; Stewart *et al.*, 2009). Its wide geographical range suggests opportunities for isolation and differentiation of populations. However, *M. sinensis* is self-incompatible and has wind-dispersed pollen and seed which are traits that would be expected to limit the differentiation of populations. Early population genetic studies with molecular markers evaluated accessions of *M. sinensis* over some of its native range, for example, in Japan (Shimono *et al.*, 2013), the Izu Islands of Japan (Iwata et al., 2005, 2006), Taiwan and the Ryukyu Islands (Chou *et al.*, 2000), South Korea (Qin *et al.*, 2013), and China (Xu *et al.*, 2013; Zhang *et al.*, 2013; Zhao *et al.*, 2013), and between South Korea and Japan (Slavov *et al.*, 2013). More recently, many accessions of *M. sinensis* from most of its native range in Japan, China, and South Korea were evaluated with restriction site-associated DNA sequencing (RAD-Seq) single nucleotide polymorphism (SNP) markers, GoldenGate SNPs, and ten plastid microsatellite markers (Clark *et al.*, 2014).

Flowering time is a crucial factor governing regional and seasonal adaptation; in addition, it is also a key target trait for extending the vegetative phase of *Miscanthus* to improve biomass potential. *M. sinensis* from high latitudes and high altitudes flower earlier than those from low-altitude and low-latitude regions in Japan, with the earliest *M. sinensis* flowering two months before the latest *M. sinensis* (Adati, 1958; Anzoua *et al.*, 2015). The genetic mechanisms for the regulation of flowering time have been characterized in many plants. *CONSTANS* (*CO*) and *Heading Date 1* (*Hd1*) are central regulators for the flowering pathway in *Arabidopsis* and rice, respectively (Valverde, 2011). *CO* and *Hd1* are orthologues that have diverged from a common ancestral gene and encode a nuclear protein that contains a CCT (CONSTANS, CO-like, and TOC1) motif and two B-box-type zinc-finger domains. The CCT motif includes a nuclear import signal (Robson *et al.*, 2001), and B-boxes are considered to be involved in protein–protein interaction rather than in DNA-binding functions (Khanna *et al.*, 2009). *CO* promotes flowering only under long photoperiods, whereas *Hd1* suppresses flowering under long-day conditions and promotes it under short-day conditions, showing bi-functionality to the photoperiodic response (Yano *et al.*, 2000; Shrestha *et al.*, 2014). In addition, in sorghum, which is closely related to *Miscanthus*, two floral activators, *SbCO* and *SbEhd1* function in photoperiod-sensitive flowering (Yang *et al.*, 2014). *SbPRR37* and *SbGhd7* repress the activity of *SbCO* and *SbEhd1*, respectively, and delay flowering in long-days (Murphy *et al.*, 2011, 2014; Yang *et al.*, 2014).

This is the first report to characterize flowering genes in the genus *Miscanthus*. Sequences of *CO*/*Hd1* homologues were compared using a broad geographical sampling from Japan, South Korea, and China. In this study, it is shown that multiple homologous copies of *CO*/*Hd1* are a common feature in *Miscanthus*. Furthermore, it was also shown that many non-functional alleles were detected in accessions from the Japanese archipelago, whereas non-functional alleles were not observed in most accessions from the Asian mainland. The factors involved with such a striking geographical difference are discussed.

## Materials and methods

### Plants

The accessions studied and their provenances are shown in Table 1. Genetic data were obtained from 44 *Miscanthus* genotypes, including 24 wild *M. sinensis* accessions collected from throughout Japan, 14 *M. sinensis* from throughout China, three *M. sinensis* from Korea, one *M. sinensis* ssp. *condensatus* from Japan, one *M. floridulus* from Japan, and one diploid *M. sacchariflorus* ‘Robustus’, and one tetraploid *M. sacchariflorus* from Japan. Subsets of accessions were phenotypically evaluated in two randomized complete block design field trials conducted at the Experiment Farm of the Field Science Center for Northern Biosphere, Hokkaido University (43°04’ N, 141°20’ E), in Sapporo, Japan. The first field trial had two replications and the second field trial had four replications. Seeds of *Miscanthus* were sown in March of 2007 in a greenhouse and seedlings of 14 Japanese *M. sinensis* accessions were transplanted in the field in June to establish the first field trial (Table 1). A second field trial was established at Hokkaido University from clonal divisions in June 2012 with nine of the Japanese accessions in the first field trial plus two additional Japanese accessions, one from Korea, and seven from China (Table 1). Accessions not included in the field trial were grown in a greenhouse.

**Table 1.** Plant materials and their characteristics

Map no.	Acc. no.	Alleles identified in *Hd1* homologues and putative loci								No. of sequenced plasmid clones	Species of genus *Miscanthus*	Prefectures (cities)	Country	Latitude	Longitude	First appearance of heading in Sapporo	
		*Hd1a*					*Hd1b*									2012	2014
1	JM0015-5	**F**	**F**	**M1**						17	*M. sinensis*	Hokkaido (Fukagawa)	Japan	43.69	142.08	22 Jul	–
2	JM0080-4	**F**	**M1**							10	*M. sinensis*	Hokkaido (Bihoro)	Japan	43.66	144.25	22 Jul	–
3	JM0079-2	**F**	**F**	**M1**	**N**					12	*M. sinensis*	Hokkaido (Iouzan)	Japan	43.62	144.45	26 Jul	28 Jul
4	JM0079-5	**F**	**F**	**M1**	**N**		**M2**			13	*M. sinensis*	Hokkaido (Iouzan)	Japan	43.62	144.45	–	–
5	JM0047-5	**F**	**F**	**M1**						15	*M. sinensis*	Hokkaido (Shakotan)	Japan	43.30	140.55	14 Aug	18 Aug
6	JM0058-1	**F**	**R**				**M2/4**			10	*M. sinensis*	Hokkaido (Toyoura)	Japan	42.60	140.65	21 Aug	27 Aug
7	JM0058-2	**F**	**F**	**N**	**N**					9	*M. sinensis*	Hokkaido (Toyoura)	Japan	42.60	140.65	3 Sep	–
8	JM0071-1	**F**	**F**	**N**						8	*M. sinensis*	Hokkaido (Kaminokuni)	Japan	41.79	140.08	8 Aug	–
9	JM0071-5	**F**	**M1**							10	*M. sinensis*	Hokkaido (Kaminokuni)	Japan	41.79	140.08	21 Aug	–
10	JM0069-4	**F**	**F**	**F**			**M2**			16	*M. sinensis*	Hokkaido (Matsumae)	Japan	41.40	140.22	–	14 Aug
11	JM0085-5	**F**	**F**	**M1**	**N**					11	*M. sinensis*	Iwate (Morioka)	Japan	39.75	141.14	21 Aug	25 Aug
12	JM0190-5	**F**	**F**	**M1**	**M1**	**N**				16	*M. sinensis*	Fukushima (Fukushima)	Japan	37.53	140.11	–	–
13	JM0091-2	**F**	**F**	**M1**	**M1**	**N**				21	*M. sinensis*	Tochigi (Nasu)	Japan	36.92	139.92	21 Sep	3 Sep
14	JM0382-5	**F**	**M1**	**M1**						15	*M. sinensis*	Gunma (Gunma)	Japan	36.81	139.31	–	–
15	JM0094-2	**F**	**M1**							10	*M. sinensis*	Nagano (Matsubarako)	Japan	36.05	138.46	21 Aug	8 Sep
16	JM0098-5	**F**	**F**	**M1**	**M1**					15	*M. sinensis*	Yamanashi (Akeno)	Japan	35.78	138.48	–	8 Sep
17	JM0273-5	**F**	**N**				**M2**			12	*M. sinensis*	Shimane (Shimane)	Japan	34.87	132.06	–	–
18	JM0118-1	**F**	**N**				**M2**			12	*M. sinensis*	Tokushima (Shiozuka)	Japan	33.94	133.68	27 Sep	26 Sep
19	JM0119-5	**R**	**N**				**M2**			8	*M. sinensis*	Kochi (Kochi)	Japan	33.56	133.38	27 Sep	27 Sep
20	JM0125-1	**F**	**N**	**N**			**M2/3**			16	*M. sinensis*	Miyazaki (Miyakonojo)	Japan	31.53	131.24	17 Oct	non
21	JM0137-4	**F**	**M1**							15	*M. sinensis*	Kagoshima (Tanegashima)	Japan	30.44	131.03	–	–
22	Uruma1b	**F**	**N**							8	*M. sinensis*	Okinawa (Uruma)	Japan	26.34	127.85	–	–
23	Onna1a	**F**	**N**				**M2**			14	*M. sinensis*	Okinawa (Onna)	Japan	26.45	127.81	–	–
24	PMS-436	**R**	**R**	**F**	**F**					13	*M. sinensis*	Liaoning (Benxi)	China	41.32	123.74	–	27 Aug
25	KMS-149	**R**	**R**	**F**						11	*M. sinensis*	Kangwon (Inje)	Korea	38.22	128.37	–	–
26	PMS-164	**R**	**R**	**F**			**M2**			12	*M. sinensis*	Hebei (Xingtai)	China	37.34	114.28	–	17 Sep
27	KMS-239	**R**	**F**				**M2**			14	*M. sinensis*	Gyeongsangbuk (Bonghwa)	Korea	36.91	128.84	–	15 Sep
28	PMS-161	**R**	**R**	**F**			**M2**			14	*M. sinensis*	Shanxi (Qinshui)	China	35.72	112.32	–	–
29	KMS-092	**R**	**F**				**M2**			9	*M. sinensis*	Jeollanam (Haenam)	Korea	34.63	126.63	–	–
30	PMS-144	**R**	**F**							8	*M. sinensis*	Shanxi (Baoji)	China	34.24	106.93	–	5 Sep
31	PMS-134	**R**	**F**	**F**						13	*M. sinensis*	Gansu (Longnan)	China	33.76	105.80	–	27 Aug
32	PMS-088	**R**	**F**	**F**						7	*M. sinensis*	Hubei (Shenlongjia)	China	31.49	110.37	–	–
33	PMS-007	**R**	**M5**	**M5**			**M2**			14	*M. sinensis*	Hubei (Badong)	China	30.80	110.26	–	13 Sep
34	PMS-303	**F**	**F**	**F**			**M2**	**M2**		12	*M. sinensis*	Zhejiang (Fuyang)	China	30.10	119.94	–-	-
35	PMS-425	**R**	**F**	**F**			**M2**			12	*M. sinensis*	Hubei (Chibi)	China	29.72	113.85	–	31 Oct
36	PMS-285	**R**					**M2**			11	*M. sinensis*	Anhui (Huangshan)	China	29.64	118.16	–	2 Oct
37	PMS-227	**F**	**F**	**F**						13	*M. sinensis*	Guizhou (Guiyang)	China	26.61	106.75	–	–
38	PMS-397	**R**	**F**	**F**	**F**					8	*M. sinensis*	Guangxi (Liuzhou)	China	24.40	109.98	–	–
39	PMS-351	**F**	**F**							8	*M. sinensis*	Guangdong (Wuhua)	China	24.07	115.63	–	–
40	PMS-381	**F**	**F**							11	*M. sinensis*	Hainan (Wuzhishan)	China	19.01	109.65	–	–
41	Mfl15-2	**R**	**F**	**F**	**N**		**N**	**N**		23	*M. floridulus*	Shimane (Masuda)	Japan	34.74	131.87	–	–
42	JM0208-5	**F**	**F**				**N**	**N**	**N**	25	*M. sinensis ssp. condensatus*	Tokyo (Hachijoujima)	Japan			–	–
43	Msa(2x)	**F**					**F**	**N**		14	*M. sacchariflorus* 'Robustus'		Japan			–	–
44	Msa(4x)	**F**	**F**	**F**	**F**	**F**	**N**	**N**	**N**	24	*M. sacchariflorus*	Miyazaki (Miyakonojo)	Japan			–	–

: Functional alleles : Non-functional alleles. F, functional alleles; R, revertant alleles; N, non-functional alleles; M1 to M5, insertion alleles of *MsiMITE1* to *MsiMITE5*

M2/M3 or M2/M4 represents having both of *MsiMITE2/MITE3* or *MsiMITE2/MITE4*. Map no. 1 to 40 corresponds to the number on the map in Fig. 4.

### Heading date

The date of first heading in the field trials was recorded weekly during the growing season; heading date was recorded and analysed instead of flowering time because the former was more consistent than the latter in our short-season location (data not shown). Heading was defined as the flower stalk emerging from the leaf sheath by ≥1cm. The 2012 heading date data was from the first field trial established in 2007, and the 2014 data was from the second trial established in 2012 (Table 1).

### DNA and RNA extraction and cDNA synthesis

Genomic DNA of 28 *Miscanthus* accessions (nos. 1–21, 25, 27, 29, and 41–44 in Table 1) was extracted from 200mg of adult leaves using the DNeasy plant DNA extraction kit (Qiagen, Tokyo, Japan) according to the manufacturer’s instructions. The remaining genomic DNA of 16 accessions was provided by the University of Illinois. Total RNA (JM0079-2 and JM0125-1) was prepared from 200mg of adult leaves using the TRIzol reagent (Life Technologies Japan Ltd., Tokyo, Japan). One microgram of total RNA was used to synthesize oligo dT primed cDNA at 50 °C for 30min using the TaKaRa AMV kit (TaKaRa Bio Inc., Shiga, Japan). This cDNA was amplified by PCR with a forward primer (5’-GATCAGGAGAAGACATATACACCC-3’) for determining the sites of splicing and terminal end in the transcript.

### DNA amplification and sequencing

Primers used in gene cloning were designed based on *CO*/*Hd1* homologues in sorghum, the closest relative of *Miscanthus* with an annotated genome. Version 1.4 of the *Sorghum bicolor* genome was queried using a BLAST search at Phytozome (http://www.phytozome.net/) and the nucleotide region detected with about 2kb on chromosome 10 was used for primer design. For 5’ and 3’ untranslated regions, the TaKaRa LA PCR *in vitro* Cloning Kit (TaKaRa) was used to obtain DNA fragments. Finally, DNA fragments (2–2.5kb) containing the 5’ flanking region to 3’ flanking region were amplified using specific primers (F14: 5’- GATCAGGAGAAGACATATACACCC-3’ and R17: 5’- GATCACTGACCACTCATGGTATAAC-3’) and Taq polymerase (TaKaRa). PCR conditions were as follows: 30 s at 96 °C, 30 s at 50 °C, and 3min at 72 °C (30 cycles). The buffer and Mg^2+^ concentration were prepared according to the user’s manual. PCR products containing the coding region were cloned into a pGEM vector (Promega KK., Tokyo, Japan). In total, seven to 25 plasmid clones of each accession were sequenced (Table 1). The nucleotide sequence was determined in both directions using an ABI 3130 genetic analyser (Life Technologies, Carlsbad, USA) with a BigDye Terminator Cycle Sequencing Kit v3.1 and then aligned with ATGC software (GENETYX Co., Tokyo, Japan). The DNA sequences obtained are available from DDBJ (http://www.ddbj.nig.ac.jp/index-e.html) with the accession numbers AB973600 to AB973675 and LC010137 to LC010211.

### Phylogenetic analysis

Phylogenetic trees were constructed using GENETYX software, version 10.1.4 (GENETYX Co, Tokyo, Japan). The Neighbor–Joining (NJ) method (Saitou and Nei, 1987) was conducted with Kimura’s (1980) two-parameter distances, ignoring indels. The corresponding sequences of sorghum were used as an out-group.

## Results

### Heading date

**Fig. 1.**
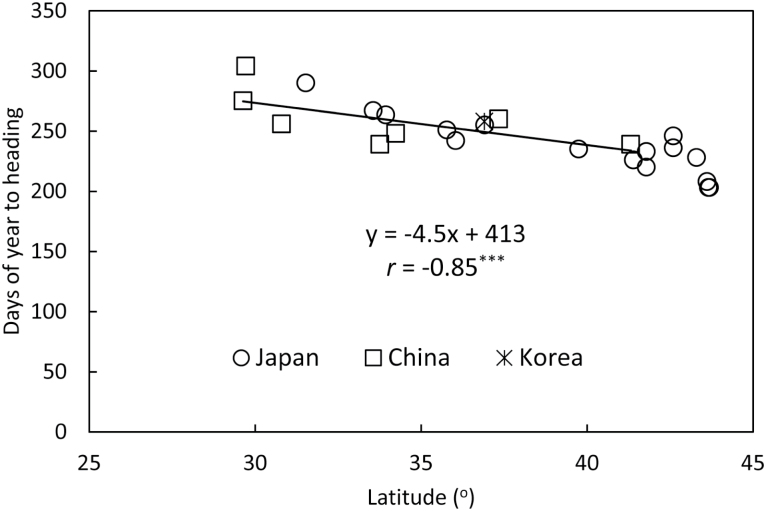
Correlation between average heading date at Sapporo in 2012 and 2014 and latitude of origin for *Miscanthus sinensis* accessions collected from the wild in Japan (open circle), South Korea (star) and China (open square).

For the subset of Japanese accessions that were evaluated for heading date in both years, the correlation between years was *r*=0.889 indicating that heading date is quite stable from year to year. A significant correlation (*r*= –0.853) was observed between latitude and heading date (Fig. 1). As expected from previous studies, there was a latitudinal gradient of heading date for both the accessions from mainland Asia and for those from Japan, with northern accessions heading earlier in the growing season than southern ones when grown in a common garden in Sapporo (Table 1). There was no latitudinal difference in flowering time between Asian mainland and Japanese accessions (Table 1; Fig. 1).

### Isolation of *MsiHd1* homologues

**Fig. 2.**
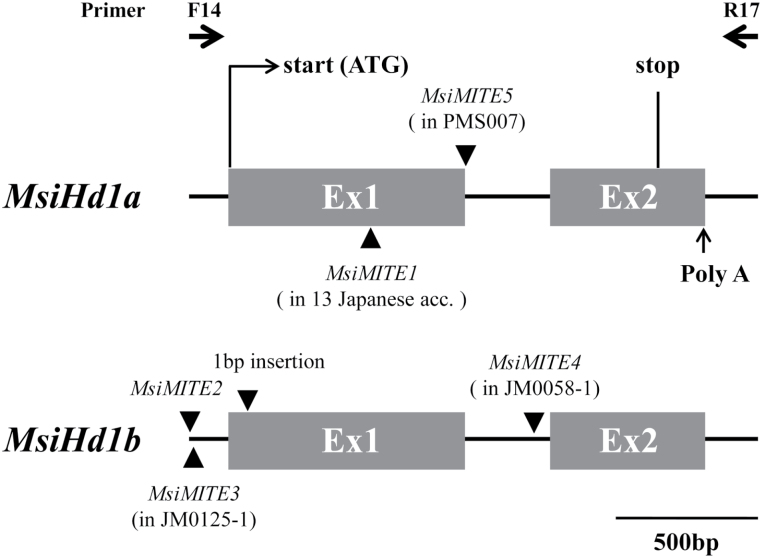
The gene structure of *Miscanthus CO/Hd1* homologues, *MsiHd1*. Two types of homologues, *MsiHd1a* and *MsiHd1b,* were detected. *MsiHd1b* is a putative pseudogene, given that its sequences are discriminated unambiguously from *MsiHd1a* by a common *MsiMITE2* insertion and additional indels with deleterious mutations, including one crucial, 1bp insertion that causes a premature stop codon. The positions of the start and stop codon and polyA addition site are shown. Insertional sites of five miniature inverted transposable elements (MITEs) (*MsiMITE1–5*) are indicated by filled triangles. *Miscanthus* accessions in which MITE sequences were detected are indicated in parentheses below the names of the MITEs. All PCR products from the genus *Miscanthus* in this study were amplified by a pair of primers, F14 and R17, depicted as arrowheads above the figure.

To isolate *CO/Hd1* homologues of *M. sinensis*, PCR amplification was carried out for 23 accessions derived from the Japanese archipelago, three accessions from South Korea, and 14 from China. Specific 2–2.5kb PCR products were amplified from all accessions. Based on the results of RT-PCR, the *M. sinensis CO*/*Hd1* sequence consisting of two exons and one intron in the PCR fragment was identified and named as *MsiHd1* (Fig. 2). The *MsiHd1* encoded approximately 400 amino acids with two conserved B-box zinc finger domains, which may be responsible for protein–protein interactions (Ben-Naim *et al.*, 2006; Wenkel *et al.*, 2006), and a CCT domain, which is involved with nuclear localization signal (Robson *et al.*, 2001) (see Supplementary Fig. S1 at *JXB* online). Both domains are features of *CO*/*Hd1*. Splicing sites of *MsiHd1* matched to those of sorghum and rice. PolyA addition sites were 159bp downstream of the stop codon (Fig. 2).

### Identification of alleles and analysis of gene phylogenetic tree

**Fig. 3.**
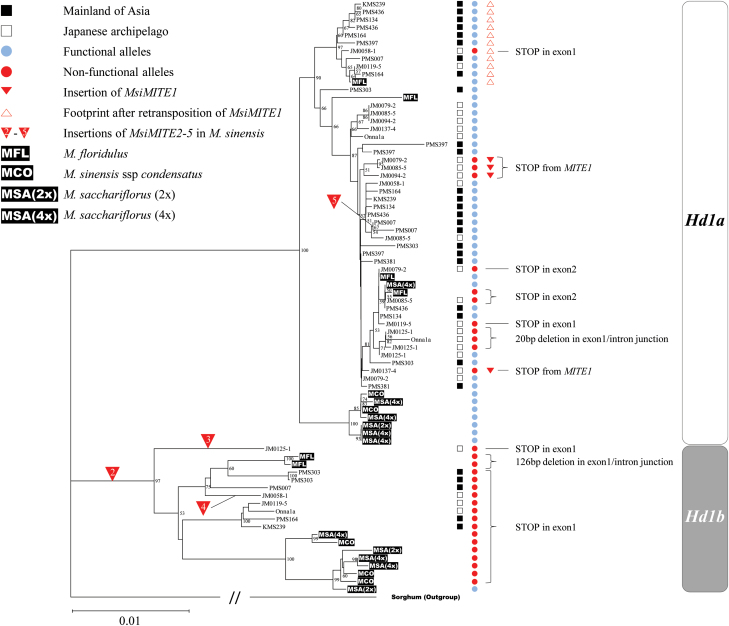
Neighbor–Joining (NJ) tree showing the relationships among 54 *CO/Hd1* alleles of 16 accessions of *Miscanthus sinensis*, six *CO/Hd1* alleles from one of *M. floridulus*, five *CO/Hd1* alleles from one of *M. sinensis* spp. *condensatus*, three *CO/Hd1* alleles from one of *M. sacchariflorus* (2×), and eight *CO/Hd1* alleles from one of *M. sacchariflorus* (4x). To facilitate viewing this figure on a single page, only about half of the accessions analysed are shown here (see Supplementary Fig. S2 at *JXB* online for the complete tree). Sixteen accessions of *M. sinensis* that represent a range of *MITE* insertions and latitudes of origin were included in this figure. *Sorghum bicolor* was used as an out-group. The phylogenetic tree was split largely into two clades, which were classified as two loci, *Hd1a* and *Hd1b*. All detected alleles of *Hd1b* in *M. sinensis* were non-functional. Major mutations that caused loss-of-function are shown to the right of the red circles, which represent non-functional alleles. The double slash on the out-group branch indicates shortening of the branch length by approximately half. Bootstrap values for nodes supported in >50% of 1000 bootstrap replicates are shown. The unnumbered squares, circles, and triangles represent the geographic origin of accessions, putative gene function, and the existence of *MsiMITE1*, respectively. The numbered triangles show *MsiMITE2* to *MsiMITE5*.

Two to five different alleles of *MsiHd1* were found in each accession, indicating that *MsiHd1* consists of at least three loci in the *Miscanthus* genome (Table 1). Based on nucleotide sequences, alleles could be assigned to two broad groups: putative functional alleles with an open reading frame and putative loss-of-function alleles caused by the same deleterious, 1bp insertion that resulted in a premature stop codon in exon 1 (Figs 2, 3).

**Fig. 4.**
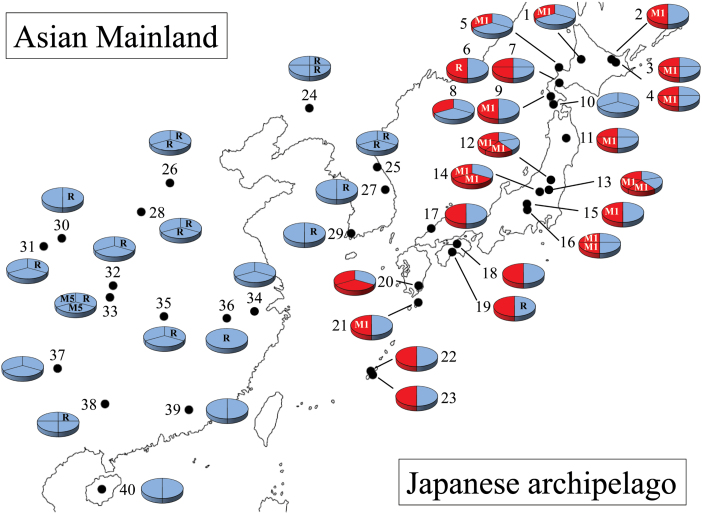
Geographic distribution of *Hd1a* alleles in *M. sinensis* accessions from East Asia. Information regarding *MsiHd1b* was omitted for simplification. The site numbers on the map correspond to map numbers in Table 1. Pie charts with one to five pieces represent the number of detected alleles in *MsiHd1a*. Blue indicates functional alleles and red indicates non-functional alleles. R, revertant allele from *MsiMITE1*; M1, *MsiMITE1*; and M5, *MsiMITE5*.

An NJ tree indicated that at least one of the multiple *MsiHd1* loci was a pseudogene locus with deleterious mutations, forming a distinctive monophyletic clade (Fig. 3; see Supplementary Fig. S2 at *JXB* online). The pseudogene locus was named as *MsiHd1b* and the others were considered alleles of the *MsiHd1a* multi-locus family. All 46 alleles of *MsiHd1a* detected in accessions from China and South Korea appeared to be functional based on their sequence, whereas about half of the alleles of *MsiHd1a* from Japanese accessions had putative loss-of-function mutations (35 functional alleles/68 total alleles=51.5%). Thus, a strikingly large difference was observed in the number of putative functional alleles between accessions from the Asian mainland and the Japanese archipelago (Fig. 4), although no clear difference for *Hd1a* alleles was observed among northern, middle, and southern latitudinal regions.

### Identification of MITE transposon and revertant alleles

**Fig. 5.**
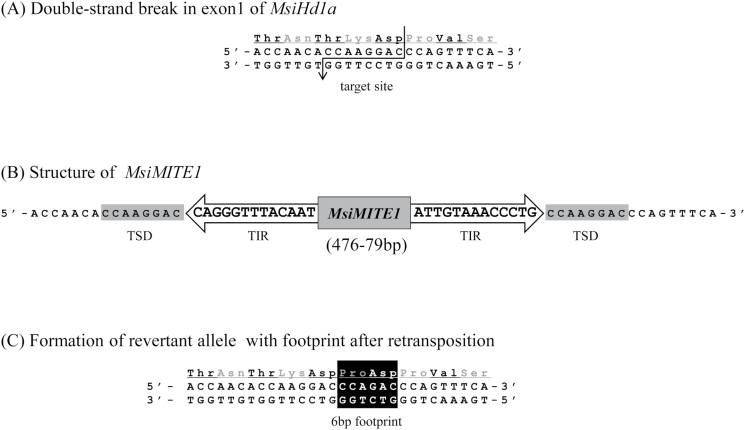
The structure of *MsiMITE1* and a schematic diagram of its insertion and retransposition. (A) A target site before insertion of *MsiMITE1*. (B) Miniature inverted transposable element (MITE)-like sequences are recognized by terminal inverted repeats (TIRs) and target site duplications (TSDs). (C) Revertant alleles contain a 6bp footprint which encodes two additional amino acids (proline and aspartic acid) at the insertion-retransposition site of *MsiMITE1*.

Five novel types of miniature inverted transposable elements (MITEs) with distinctive terminal structures (TIRs: terminal inverted repeats, TSDs: target site duplications) were identified in *MsiHd1* alleles and named as *MsiMITE1*–*MsiMITE5* (Fig. 5; see Supplementary Fig. S3 at *JXB* online). *MsiMITE1* and *MsiMITE5* were found in *MsiHd1a*, whereas the other three MITEs were found in *MsiHd1b* (Table 1; Figs 2, 3). MITEs are known to be preferentially located in the vicinity of genes (Bureau and Wessler, 1994; Feschotte *et al.*, 2002). During the alignment work, if a relatively large insertion gap (<500bp) was found and both ends of the gap had TIRs and TSDs, the insertion sequence was identified as a MITE. *MsiMITE1*, which was only detected in Japanese accessions, was found to cause loss-of-function of *MsiHd1a* at the insertion site by forming a stop codon. On the other hand, many accessions from the Asian mainland had revertant alleles derived from retransposition, leaving a footprint of 6bp instead of a full *MsiMITE1* sequence (Fig. 5). Revertant alleles were found in most accessions from the Asian mainland but in only one Japanese accession (JM0119-5; although another Japanese accession, JM0058-1, also had a footprint of 6bp, that allele was considered not to be a revertant allele because it had an additional putative loss-of-function mutation; Table 1). Two MITE subfamilies (*MsiMITE2* and *MsiMITE3*) were observed in tandem in the 5’ untranslated region of the *MsiHd1b* locus (Fig. 2). *MsiMITE3, MsiMITE4*, and *MsiMITE5* were only detected in JM0125-1, JM0058-1, and PMS007, respectively. These three MITEs were also detected in other Poaceae species (see Supplementary Fig. S4 at *JXB* online), but *MsiMITE1* and *MsiMITE2* were not (see Supplementary Fig. S3 at *JXB* online).

### Variation in the number of copies of *MsiHd1* among *Miscanthus* species

Specific PCR products of *CO*/*Hd1* homologues were also obtained from other *Miscanthus* species including *M. sinensis* ssp. *condensatus*, *M. floridulus*, *M. sacchariflorus* (2x), and *M. sacchariflorus* (4x) and their nucleotide sequences were determined. In total, 76 unique sequences (alleles) were obtained from *Miscanthus* genomes (Table 1; Fig. 3). Similar to *MsiHd1a* and *MsiHd1b* in *M. sinensis*, *MflHd1a/b* in *M. floridulus*, *McoHd1a*/*b* in *M. sinensis* ssp. *condensatus*, *Msa (2x) Hd1a/b* in *M. sacchariflorus* (2x), *Msa (4x) Hd1a/b* in *M. sacchariflorus* (4x) were identified and named, respectively. A functional allele at *Hd1b* was detected only in *M. sacchariflorus* (2x). In *Hd1b* of both *M. sinensis* ssp. *condensatus* (*McoHd1b*) and *M. sacchariflorus* (4x) (*Msa(4x)Hd1b*), three non-functional alleles were detected, consisting of at least two loci in which pseudogenization occurred. Five alleles of *Msa(4x)Hd1a* were detected from *M. sacchariflorus* (4x). *M. floridulus* had a revertant allele with a 6bp footprint, however, *MsiMITE1*-like sequences could not be detected from the *M. floridulus* genome.

## Discussion

Unexpectedly, among *M. sinensis* accessions from a broad latitudinal distribution, no correlation between days to heading and the polymorphisms of *MsiHd1* was observed. However, a clear difference in the distribution of the putative functional genes in *MsiHd1a* was observed between accessions from the Asian mainland and those from the Japanese archipelago (Fig. 4; Table 1). This difference shows that dependency on the gene function of *MsiHd1a* is different between the two areas. In *Miscanthus*, the presence or absence of other flowering-time genes epistatic to *Hd1* might have promoted the geographic allelic differentiation of *MsiHd1a* between populations from the Asian mainland and the Japanese archipelago. Similar patterns of differentiation of flowering-time genes have been observed in other plant species. For example, *FRIGIDA (FRI*) in *Arabidopsis* enhances the effect of *FLOWERING LOCUS C (FLC),* which is downstream of *FRI* in the floral pathway, and thus only accessions with functional alleles of *FRI* are subjected to the influence of *FLC* alleles for the number of days to flowering (Caicedo *et al.*, 2004). In addition, in a field experiment with *Arabidopsis*, a correlation between the number of days to flowering and the latitude of origin was observed only among accessions with functional *FRI* alleles, and accessions from lower latitudes flowered earlier (Stinchcombe *et al.*, 2004). Since no correlation was observed among accessions with non-functional *FRI*, it seems that geographic differentiation of *FLC* alleles in *Arabidopsis* depends on the epistasis (gene interaction) of *FLC* and *FRI*. In rice, much research on the epistasis of *Hd1* to other flowering-time genes has been reported (Lin *et al.*, 2000; Uwatoko *et al.*, 2008; Takahashi *et al.*, 2009; Ito and Izawa, 2013; Chen *et al.*, 2014). Rice *Hd6,* which encodes a protein kinase CK2α, suppresses flowering only in the presence of functional *Hd1* (Takahashi *et al.*, 2001; Ogiso *et al.*, 2010). In a population structure study using neutral genetic markers, Slavov *et al.* (2013) identified two subpopulations of *M. sinensis*, with one from mainland Asia around the Korean peninsula and the other from Japan, but they also observed a similar latitudinal cline for flowering time in both subpopulations; their results are consistent with our findings. It is expected that analyses of individual genes could best explain local adaptation and the evolutionary history of *M. sinensis*. Studies on other flowering-time genes of *Miscanthus* would further elucidate the population differentiation observed in *MsiHd1a* alleles in the present study.

**Fig. 6.**
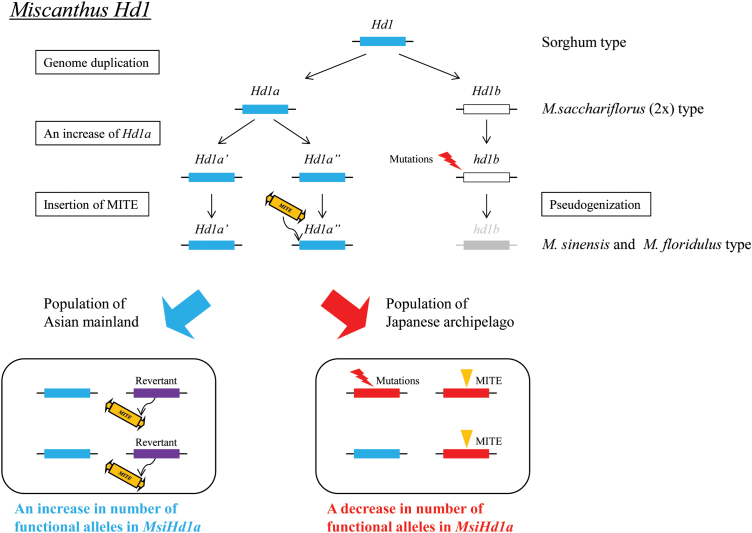
Schematic model for *Miscanthus Hd1* evolution. Blue indicates functional alleles and red indicates non-functional alleles. The whole genome duplication of *Miscanthus* resulted in *Hd1a* and *Hd1b* loci. Accumulation of deleterious mutations caused pseudogenization of *Hd1b* in *M. sinensis*. By additional local duplication, *Hd1a* increased in copy number (duplicated genes designated as *Hd1a’* and *Hd1a’’* in figure). After the last glacial maximum, the number of functional *Hd1a* alleles in the Asian mainland population of *M. sinensis* was high, whereas, in the Japanese population, the number of functional alleles decreased due to the accumulation of alleles with MITE insertions and/or other deleterious mutations. This difference suggests that dependency on the gene function of *Hd1a* is different between *M. sinensis* populations from the Asian mainland and the Japanese archipelago.

The NJ tree showed that 20 of the 44 *Miscanthus* accessions that were analysed had two diverged loci, *Hd1a* and *Hd1b* (Fig. 3; see Supplementary Fig. S2 at *JXB* online). Although their positions on the *Miscanthus* genome are unknown, the existence of duplicated *Hd1* loci can be considered to be universal within the genus *Miscanthus*, in contrast to sorghum, maize, and rice which have only one *Hd1* locus. The recent genome duplication of *Miscanthus* relative to sorghum (Kim *et al.*, 2012; Ma *et al.*, 2012; Swaminathan *et al.*, 2012) can at least partially account for differences in the number of *Hd1* loci. Large-scale genomic analyses of *M. sinensis* have revealed that *M. sinensis* (*x*=19) is a diploidized tetraploid species formed by the duplication of chromosomes after the divergence from an *x*=10 ancestor (Kim *et al.*, 2012; Ma *et al.*, 2012; Swaminathan *et al.*, 2012). The three or more loci of *MsiHd1* identified by this study in *M. sinensis* might have been caused in part by the whole genome duplication (*MsiHd1a* and *MsiHd1b*) and also by local gene duplications (multiple *MsiHd1a*) via unequal crossing-over (Fig. 6). Although gene duplication can be an evolutionary process to gain new function (Ohno, 1970), a duplicated gene can alternatively be subjected to inactivation by mutations and genetic drift (Hughes, 1994; Force *et al.*, 1999). Redundancy of gene functions among multiple *MsiHd1* loci through polyploidization might have allowed pseudogenization of *MsiHd1b*. The precise number of *MsiHd1a* loci in *Miscanthus* remains unknown. In two Japanese accessions, JM0190-5 and JM0091-2, five copies of *MsiHd1a* were detected, suggesting the possibility of additional duplications in *MsiHd1a*. The *M. floridulus Hd1* locus, having a revertant allele, showed the greatest sequence similarity to the *M. sinensis Hd1* locus (Fig. 3) of the species evaluated. *McoHd1b* and *Msa(4x)Hd1b* in *M. sinensis* spp. *condensatus* and *M. sacchariflorus* (4x), respectively, each had three alleles, suggesting at least two loci, in contrast to the putative single locus in *M. sinensis*. The observation of five alleles of *Msa(4x)Hd1a* within one individual *M. sacchariflorus* (4x) indicates that there are at least three *Msa(4x)Hd1a* loci in this individual. As observed above, copy number of *Hd1a/b* varies among species in the genus *Miscanthus*.

Differentiation of *M. sinensis* between mainland Asian populations and Japanese populations for number of functional *Hd1* alleles is consistent with Clark *et al.*’s (2014) finding that migration after the last glacial maximum from a refugium in south-eastern China to Japan occurred prior to migrations northward in mainland Asia. Moreover, Clark *et al.* (2014) estimated that the nascent Japanese *M. sinensis* population survived the Younger Dryas as a distinct group, which probably included strong natural selection for adaptation to a short growing season, whereas *M. sinensis* in mainland Asia remained in longer-season lower latitude environments until more recently. However, the phylogenetic tree of *MsiHd1* in the present study identified no distinct clear clade that separates Japan from the Asian mainland (Fig. 3). Thus, our hypothesis is that the polymorphism seen within *MsiHd1* existed in the ancestral population of *M. sinensis*, as opposed to being a recent set of mutations originating from distinct geographic regions, which is consistent with the data of Clark *et al.* (2014) that suggested a recent (14 000 years before present and later) but differential expansion of the range of *M. sinensis* throughout East Asia that allowed for a different history of selection pressure on mainland and Japanese populations. Although *M. sinensis* populations from mainland Asia and Japan currently differ in the number of functional *MsiHd1* alleles, genotypes of both populations collected from similar latitudes head at similar times when grown in a common garden, suggesting that they may have differing genetic mechanisms to arrive at the same phenotypic result. This insight leads to the hypothesis that crosses between mainland *M. sinensis* genotypes and Japanese *M. sinensis* genotypes are more likely to exhibit transgressive segregation for heading date than crosses within either geographic group, assuming similar latitudes of origin of the parents for the comparisons.

In this study, in addition to the five novel MITE-like transposable elements (*MsiMITE1–5*) that were identified in the *CO*/*Hd1* homologues of *M. sinensis*, a putative revertant allele with evidence of retransposition of *MsiMITE1* was also found (Figs 3, 5; Table 1). It is possible to identify MITEs if their basic features, including TIRs and TSDs, are determined using sequence alignments (Nagano *et al.*, 2002). Of the five detected *MsiMITEs*, three were found to have homologues with similar sequences in the genomes of sugarcane, sorghum, and switchgrass (see Supplementary Fig. S4 at *JXB* online). The TIRs and TSDs were also observed in the genomes of these other plant species, suggesting that at least these elements are shared among other plant species. Since the early 1990s, when MITEs were reported in the vicinity of the *waxy* gene of rice and maize, respectively (Umeda *et al.*, 1991; Bureau and Wessler, 1992), many more MITE families were identified in plants and animals including humans (Feschotte *et al.*, 2002). MITEs are currently ordered and classified into six superfamilies (*Tc1/mariner*, *PIF/Harbinger*, *hAT*, *Mutator*, *CACTA*, and/or *Micron*; Lu *et al.*, 2012; Han *et al.*, 2013). In these definitions, *MsiMITE1*, *MsiMITE3*, and *MsiMITE5* belong to the *hAT* superfamily (8bp of TSD, >5bp of TIR), and *MsiMITE2* and *MsiMITE4* belong to the *PIF/Harbinger* superfamily (3bp of TSD, >4bp of TIR) (see Supplementary Fig. S3 at *JXB* online). *MsiMITE1* and *MsiMITE2,* which are specifically identified within *MsiHd1a* or in the 5’ UTR of *MsiHd1b,* respectively, do not have similar sequences in any genome database. However, our Southern blot analysis using a probe spanning the full length of *MsiMITE1* (ca. 500bp) detected multiple discrete signals in the *M. sinensis* genome (see Supplementary Fig. S5 at *JXB* online). This result suggests that many unidentified *MsiMITE1* sequences exist in *M. sinensis*. It remains unclear whether the richness of MITEs observed in the region of *MsiHd1a/b* is typical of most genes in *M. sinensis*. The future accumulation of genome information for *M. sinensis* will help to address these questions.

In conclusion, the comparison of *MsiHd1* homologous sequences among accessions derived from East Asia revealed contrasting allelic distribution in *M. sinensis* from the Asian mainland and Japanese archipelago. Further studies on other flowering-time genes will be necessary to elucidate the genetic architecture of flowering time and its evolution in *Miscanthus* and to improve biomass potential by regulation of the reproductive phase.

## Supplementary data

Supplementary data can be found at *JXB* online.

Supplementary Fig. S1. Alignment of the *CO/Hd1* homologues from *Miscanthus sinensis* (a functional allele from JM0079-2), *Sorghum bicolor* and *Oryza sativa*.

Supplementary Fig. S2. Complete phylogenetic tree constructed using the Neighbor–Joining (NJ) method.

Supplementary Fig. S3. The structure of *MsiMITE2–5*.

Supplementary Fig. S4. Comparison of three miniature inverted transposable elements (MITEs) found in the genomes of *M. sinensis*, sugarcane, sorghum and switchgrass.

Supplementary Fig. S5. Comparison of Southern blotting patterns between *MsiHd1* (left panel) and *MsiMITE1* (right panel).

Supplementary Data
