# Supplementary material for: Contrasting allelic distribution of CO/Hd1 homologues in Miscanthus sinensis from the East Asian mainland and the Japanese archipelago
Source: J Exp Bot. 2015 Jun 18;66(14):4227–37. doi: 10.1093/jxb/erv292 (PMC4493791; doi:10.1093/jxb/erv292)
Supplement: Supplementary Data [file supp_66_14_4227__index.html]

Contrasting allelic distribution of CO/Hd1 homologues in Miscanthus sinensis from the East Asian mainland and the Japanese archipelago — Contrasting allelic distribution of CO/Hd1 homologues in Miscanthus sinensis from the East Asian mainland and the Japanese archipelago — Supplementary Data 

# Contrasting allelic distribution of *CO*/*Hd1* homologues in *Miscanthus sinensis* from the East Asian mainland and the Japanese archipelago

## Supplementary Data

Data files

- Supplementary Data - Supplementary Data
