# Supplementary material for: Contrasting allelic distribution of CO/Hd1 homologues in Miscanthus sinensis from the East Asian mainland and the Japanese archipelago
Source: J Exp Bot. 2015 Jun 18;66(14):4227–37. doi: 10.1093/jxb/erv292 (PMC4493791; doi:10.1093/jxb/erv292)
Supplement: Supplementary Data [file supp_erv292_jexbot148965_file001.pdf]

Contrasting allelic distribution of CO/Hd1 homologs in Miscanthus sinensis from the east Asian mainland and the Japanese archipelago.

Hironori Nagano, Lindsay V. Clark, Hua Zhao, Junhua Peng, Ji Hye Yoo, Kweon Heo, Chang Yeon Yu, Kossonou Guillaume Anzoua, Tomoaki Matsuo, Erik J. Sacks, and Toshihiko Yamada

Supplemental Files

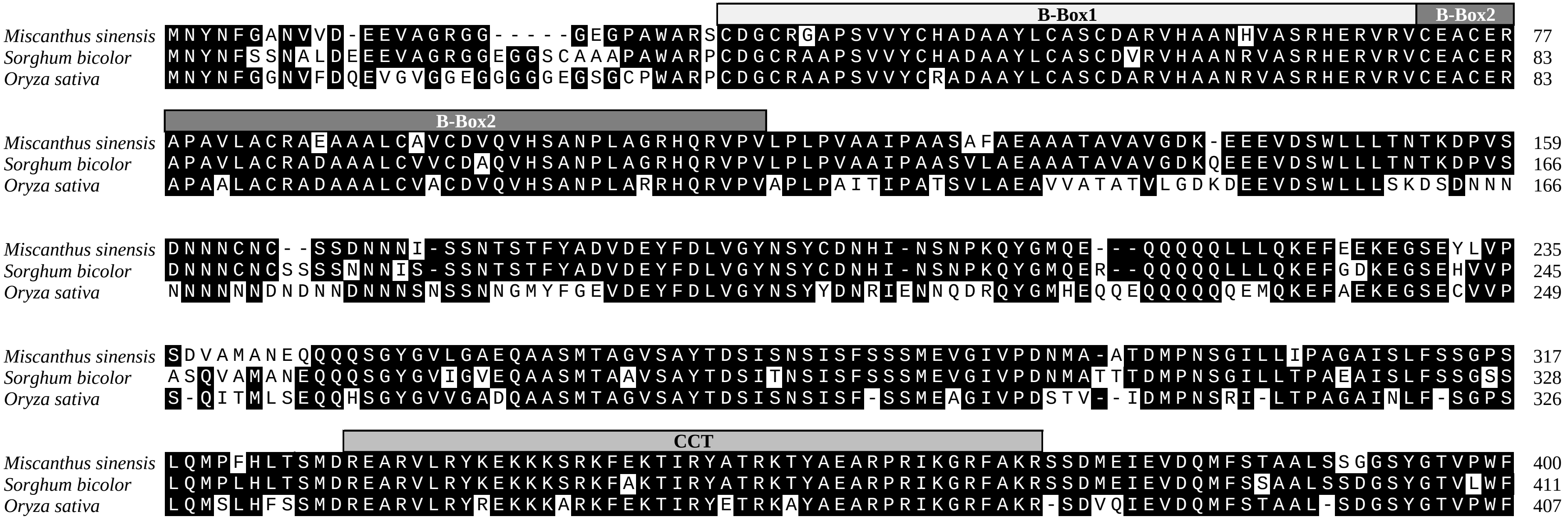

Supplementary Fig.1 Alignment of the *CO/Hd1* homologs from *Miscanthus sinensis* (a functional allele from JM0079-2), *Sorghum bicolor* and *Oryza sativa*. Amino acid sequences are available on NCBI as accession numbers AB973600, XP_002436860 and BAB17628, respectively. Highlights in black and dashes represent conserved amino acids among all three homologs and gaps in the alignment, respectively. The conserved B-Box and CCT domains, indicated with boxes above alignment were reported by Yang et al. (2014). Amino acid numbers are shown on right.

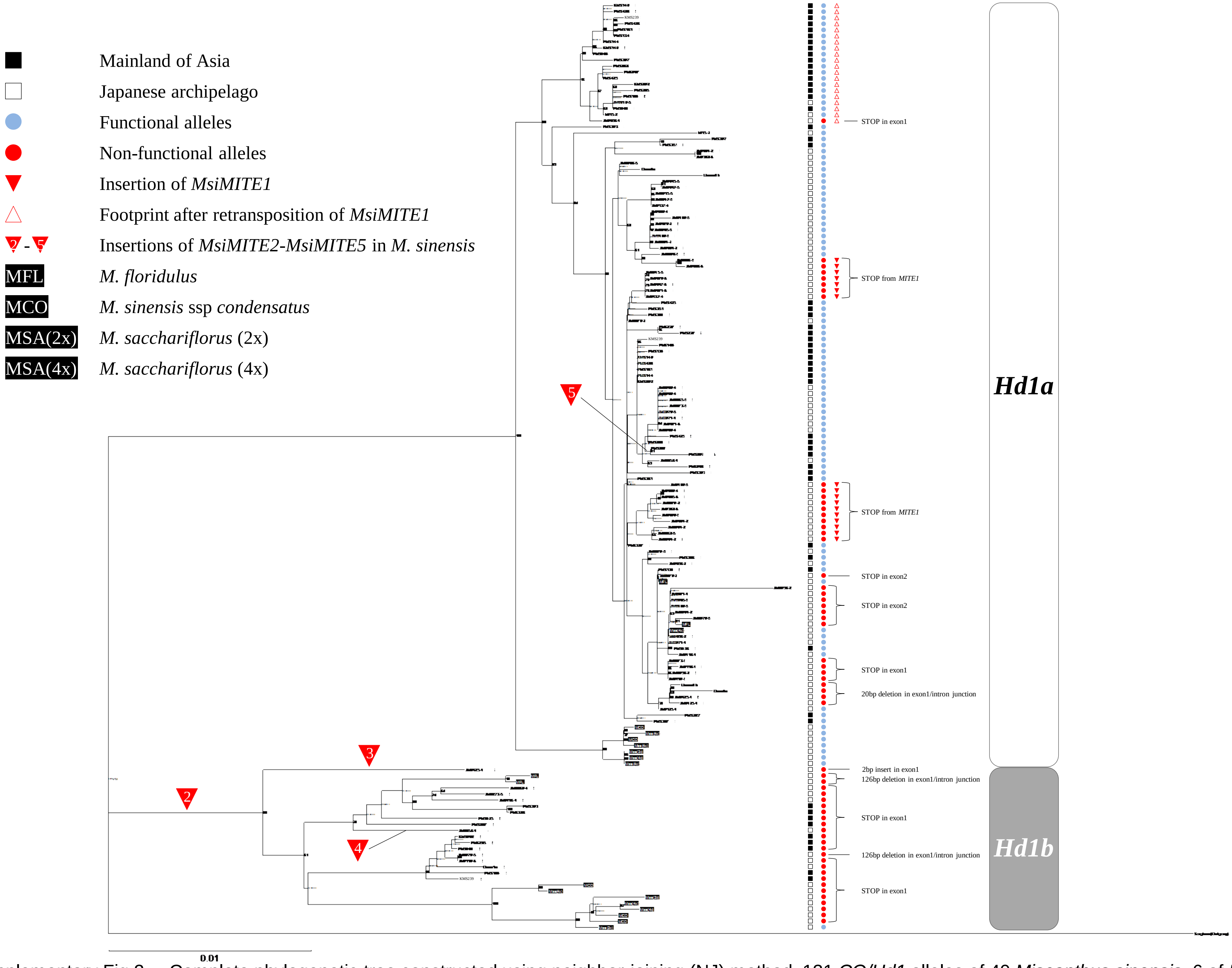

Supplementary Fig.2 Complete phylogenetic tree constructed using neighbor-joining (NJ) method. 131 *CO/Hd1* alleles of 40 *Miscanthus sinensis*, 6 of *M. floridulus*, 5 of *M. sinensis* spp. *condensatus*, 3 of *Miscanthus sacchariflorus* (2x) and 8 of *M. sacchariflorus* (4x) were analyzed.

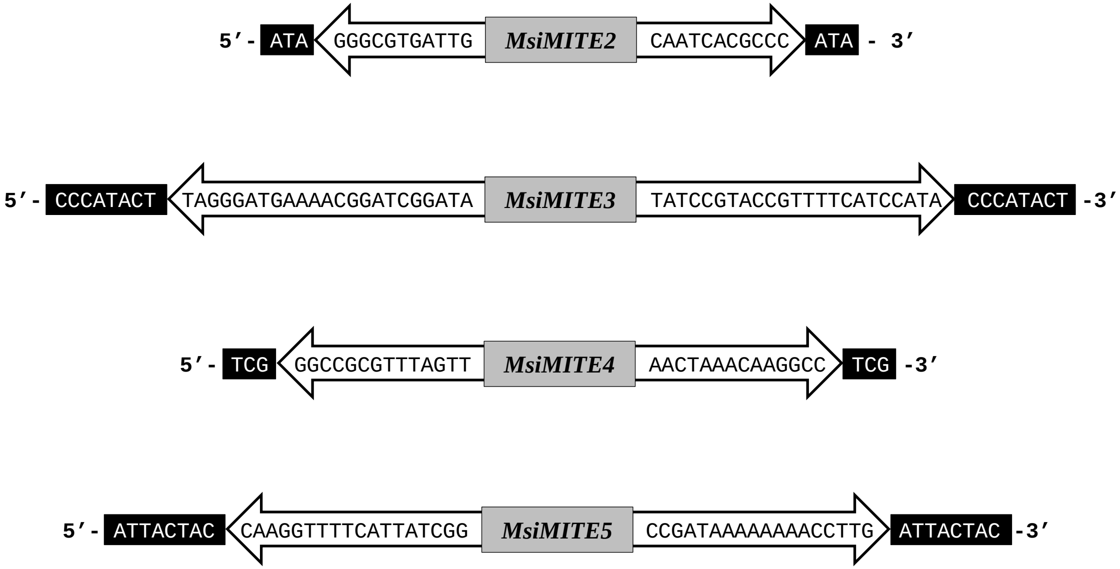

TIR (bp)	TSD (bp)	Size (bp)	Superfamily	Detected other Species (nucleotide identities(%))
11	3	350-385	*PIF/Harbinger*	None
23	8	276	*haT*	Sorghum (90%)<br>Sugarcane (89%)
14	3	274	*PIF/Harbinger*	Sugarcane (78%)
18	8	351	*haT*	Sugarcane (87%)<br>Switchgrass (83%)

Supplementary Fig.3 The structure of *MsiMITE2* to *5*. The open arrows indicate terminal inverted repeats (TIRs) and the black highlighted letters represent target site duplications (TSDs). Based on the length of TIRs and TSDs, *MsiMITE* elements were classified to two superfamilies, *PIF/Harbinger* and *hAT*. Other species in which *MITE*s similar to *MsiMITE3*, *MsiMITE4* and *MsiMITE5* were detected by BLASTN search, and the corresponding nucleotide identities are shown.

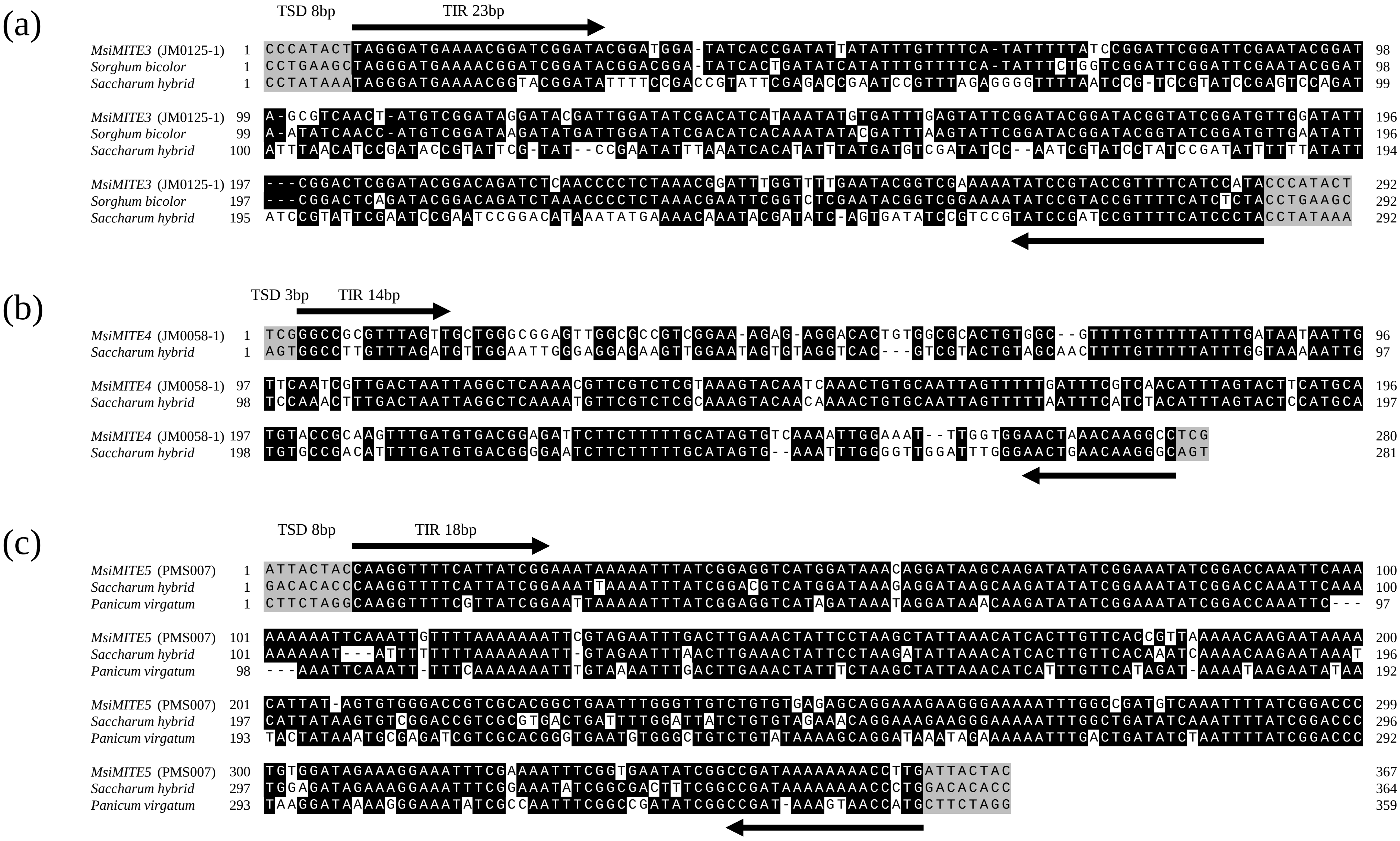

Supplementary Fig.4 Comparison of three miniature inverted transposable elements (MITEs) found in the genomes of *M. sinensis*, sugarcane, sorghum and switchgrass. Highly similar sequences were obtained from public databases using BLAST. Black background in the alignments show consensus nucleotides. The arrows indicate terminal inverted repeats (TIRs) and the gray background represents target site duplications (TSDs). Note that a pair of TSDs coincide perfectly, but they have different TSD sequences among alignments, indicating that these insertional sequences are exactly transposable elements, but not simple detecting the similar sequences among species.. Accession numbers are as follows: (a) Sorghum AF114171 (94,145–94,436bp), Sugarcane FN431663 (107,916–108,207bp) (b) Sugarcane FN431668 (65,737–65,457bp), (c) Sugarcane FN431661 (71,632–71,995bp), swichgrass AC243240 (109,072–108,714bp).

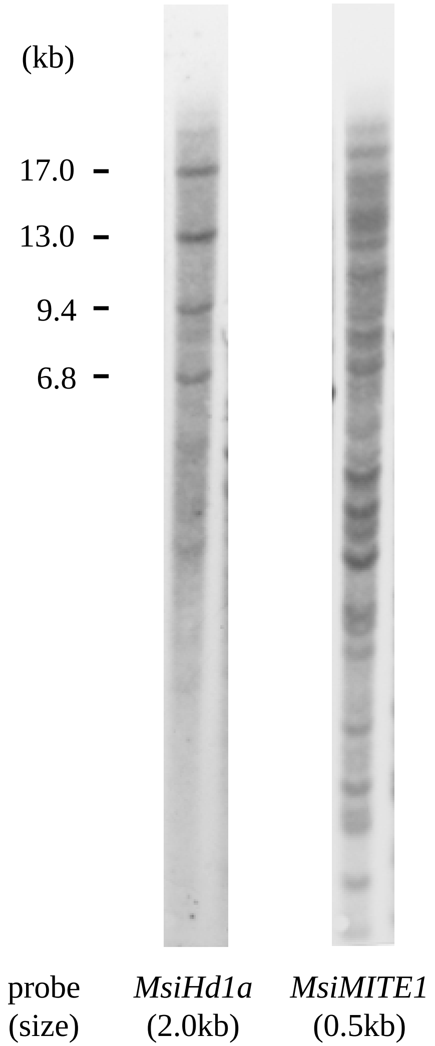

Supplementary Fig.5 Comparison of Southern blotting patterns between *MsiHd1* (left panel) and *MsiMITE1* (right panel). JM0085-5 DNA digested with *Eco*RI was run in 0.8% agarose gel electrophoresis. The DNA was transferred to a nylon membrane, and hybridization signal was detected on same membrane using labeled probes (fragments of *MsiHd1* and *MsiMITE1*, respectively) with the AlkPhos kit (GE Healthcare, UK).The membranes were washed twice in the standard condition at 55 ° C for 10 min.
